# A novel *MAP7D1* mutation causes mitotic defects and RPS14 accumulation in Shwachman−Diamond syndrome patient cells

**DOI:** 10.1242/dmm.052409

**Published:** 2025-08-26

**Authors:** Seren Kucukvardar, Arzu Karabay

**Affiliations:** ^1^ Molecular Biology-Genetics and Biotechnology, Graduate School, Istanbul Technical University, Maslak 34469, Istanbul, Turkey; ^2^ Department of Molecular Biology and Genetics, Istanbul Technical University, Maslak 34469, Istanbul, Turkey

**Keywords:** MAP7D1, Microtubule, Mitotic spindle, Shwachman−Diamond syndrome, RPS14

## Abstract

The importance of microtubule stability and microtubule-associated proteins in the etiology of Shwachman−Diamond syndrome (SDS) has been highlighted in recent studies. In one patient with SDS, a novel *MAP7D1*:c.601C>T, p.R201W variant has been identified. In this study, the causality of this variant in the pathogenesis of SDS was investigated. Mutation in the microtubule-binding domain of MAP7D1 caused disruption of its interaction with microtubules. SDS fibroblasts exhibited a decreased cell size with reduced microtubule density, and mitotic defects, including multipolar or bipolar unstable spindles, lagging chromosomes, and shortened inter-centrosomal distance. Additionally, ribosomal protein S14 (RPS14) accumulated within incorrectly dividing SDS fibroblasts. To further evaluate whether these abnormalities are directly attributable to the MAP7D1 mutation, mitotic processes were investigated through genetic manipulations of *MAP7D1* in T98G glioblastoma and HEK293T embryonic kidney cell lines. Consistent with data from SDS fibroblasts, similar phenotypes were detected upon overexpression of mutant MAP7D1 and depletion of MAP7D1. Our findings revealed that the MAP7D1 mutation acts as a loss-of-function mutation and contributes to SDS pathogenesis by disrupting microtubule dynamics and ribosomal protein regulation, identifying *MAP7D1* as a gene with substantial impact for SDS.

## Teaser

**Summary:** This study, for the first time, functionally identifies microtubule-associated *MAP7D1* as a novel causative gene for Shwachman−Diamond syndrome using patient fibroblasts and shows cell cycle-related roles of MAP7D1 protein.

## INTRODUCTION

Shwachman−Diamond syndrome (SDS) is an autosomal recessive ribosomopathy classified within hereditary bone marrow failure disorders (Shwachman et al., 1964; Narla and Ebert, 2010; Dror et al., 2011; Nelson and Myers, 2018; Warren, 2018). SDS is clinically characterized by neutropenia, exocrine pancreatic insufficiency, skeletal deformities and an elevated risk of hematological malignancies, such as myelodysplastic syndrome and acute myeloid leukemia (Dror et al., 2011; Nelson and Myers, 2018). The incidence of SDS is 1:75,000 and it generally presents in infancy (Farooqui et al., 2023), with SDS patients exhibiting a life expectancy into the third and fourth decades of life (Farooqui et al., 2023).

Almost 90% of SDS cases are associated with mutations in the SBDS ribosome maturation factor [also known as Shwachman-Bodian-Diamond syndrome (*SBDS*)] gene. Moreover, inherited variants within elongation factor-like GTPase 1 (*EFL1*), DnaJ heat shock protein family (Hsp40) member C21 (*DNAJC21*) and signal recognition particle 54 (*SRP54*) have been identified as genetic causes of SDS (Boocock et al., 2003; Nelson and Myers, 2018). Although the proteins encoded by above mentioned causal genes related to SDS are involved in maturation of the large subunit of ribosome (60S), the SBDS protein has additional functions, such as binding to microtubules, stabilizing them and localizing to mitotic spindles (Austin et al., 2008; Orelio et al., 2009). A previous study by Austin and colleagues has shown that cells of SDS patients that carry a mutation of *SBDS* divide improperly, generating multipolar spindles during mitosis, which could contribute to bone marrow failure and leukemogenesis (Austin et al., 2008). Additionally, a recent study discovered that cells of SDS patients with a mutated version of *SBDS* exhibit abnormally rapid mitosis, and lagging chromosomes were detected in a substantial percentage of cells, enhancing chromosomal instability (Sera et al., 2024). Taken together, the mitotic spindle stability and, thus, microtubule-associated proteins have important roles in the development of SDS. However, microtubule-associated proteins have not yet been investigated in the etiology of SDS.

Microtubule-associated protein 7 domain-containing 1 (MAP7D1) is a member of the MAP7 protein family, consisting of four members, i.e. MAP7, MAP7D1, MAP7D2 and MAP7D3. All family members have a highly conserved coiled-coil (cc) domain in the N-terminal region, responsible for interaction with microtubules and, in the C-terminal region, a conserved MAP7 cc domain that enables binding to the motor protein kinesin-1 (Metzger et al., 2012; Koizumi et al., 2017; Hooikaas et al., 2019; Pan et al., 2019). Thus, MAP7 proteins facilitate the transport of kinesin-dependent vesicles and organelles to the cell periphery or axon terminals by maintaining kinesin–microtubule connection (Chaudhary et al., 2019). Specifically, MAP7D1 is localized to microtubules, following a decreasing pattern from the periphery of the centrosome to the microtubule plus-ends in HeLa cervical cancer cells (Kikuchi et al., 2018; Hooikaas et al., 2019), whereas the protein is mainly localized to the centrosome and partially on microtubules within N1-E115 mouse neuroblastoma cells (Kikuchi et al., 2022). These studies show that localization of MAP7D1 in proliferating cells differs slightly, depending on cell type. In neurons, non-proliferating cells, MAP7D1 is localized to axons or the somatodendritic regions (Pan et al., 2019), involving in axon elongation, neurite length regulation, microtubule stabilization and remodeling through promotion of the Wnt5a signaling pathway (Koizumi et al., 2017; Kikuchi et al., 2018, 2022). Also, a recent study has indicated that MAP7D1 is essential in regulating the G1 cell cycle phase and the cellular response to DNA damage repair (Dullovi et al., 2023).

A male patient, whose parents were 4th-degree relatives, had been identified in a genetic study to exhibit SDS phenotypes, such as thrombocytopenia, anemia, neutropenia and exocrine pancreatic insufficiency, as well as symptoms, such as intellectual disability and insensitivity to pain (Oguz, 2020). Moreover, sequence analysis of previously reported SDS-related genes was performed in that study but interestingly, no variants were detected in these genes. Whole-exome sequencing and homozygosity mapping were conducted, and the pathogenic novel gene variant *MAP7D1*:c.601C>T, p.R201W was detected. Furthermore, the same study determined *in silico* that the *MAP7D1* variant affecting the microtubule-binding site of MAP7D1 drastically alters the protein's three-dimensional (3D) structure and tubulin-binding site of the protein in 3D modeling and docking analyses (Oguz, 2020).

In our current study, we investigated the potential causative role of the MAP7D1:p.R201W variant in SDS by impairing microtubule-associated cellular processes. We show that the MAP7D1:p.R201W mutation drastically impaired microtubule−MAP7D1 interaction, resulting in mitotic abnormalities, especially unstable multipolar or bipolar mitotic spindle assembly, shortened inter-centrosomal distances during metaphase and anaphase/telophase. This mutation also diminished the association between MAP7D1 and γ-tubulin in SDS fibroblasts. Moreover, the MAP7D1:p.R201W mutation induced accumulation of ribosomal protein S14 (RPS14) in aberrantly dividing SDS fibroblasts, suggesting that the MAP7D1 mutation contributes to SDS by impairing both microtubule-associated cellular processes and ribosomal protein regulation, similar to SBDS mutation.

## RESULTS

### MAP7D1:p.R201W mutation weakens the physical interaction between microtubules and MAP7D1 protein

**Fig. 1.**
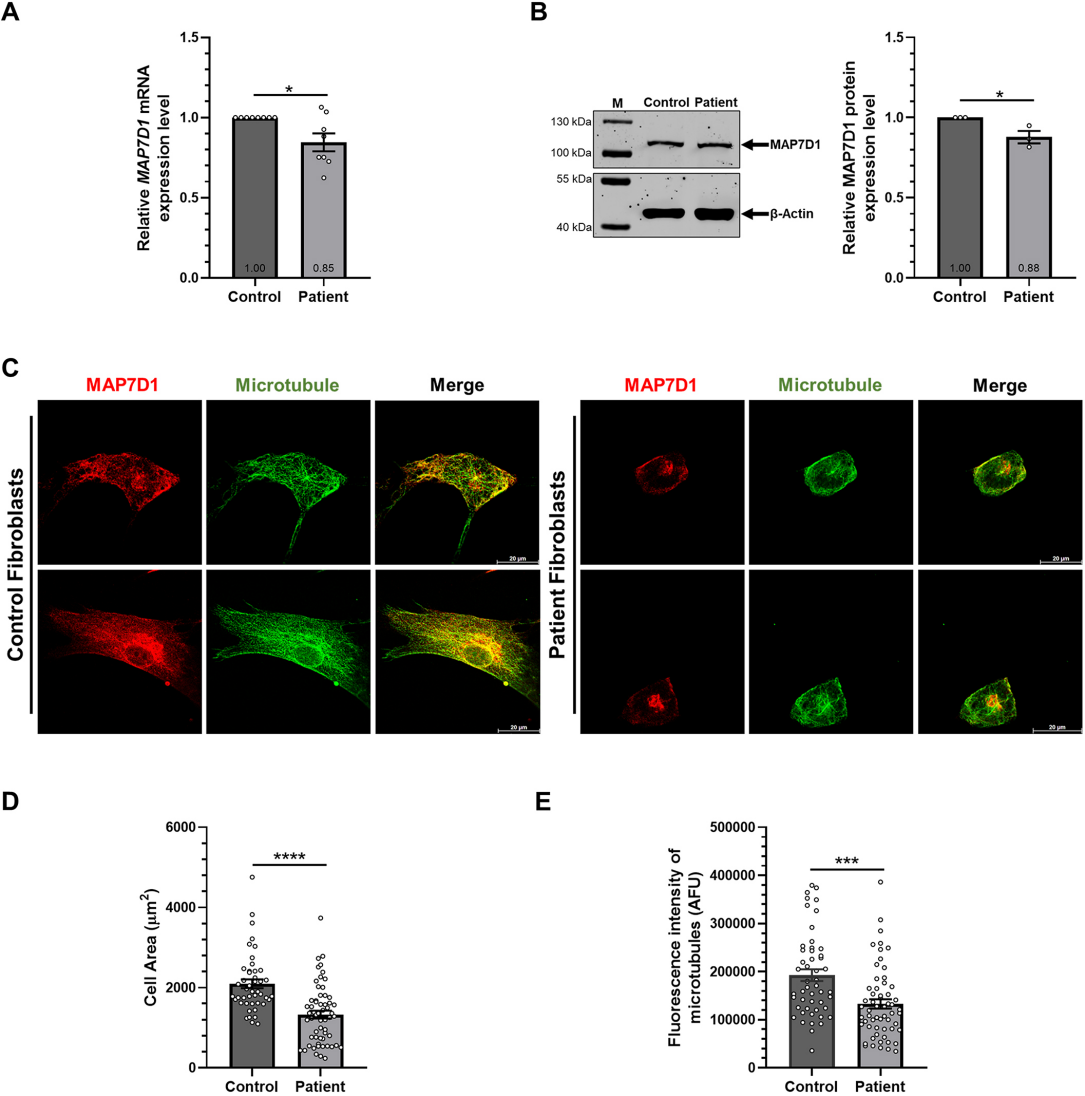
**Microtubule mass is reduced in SDS patient cells.** (A) mRNA levels of *MAP7D1* in control and patient fibroblasts. The experiment was performed in three biological replicates, each including three independent technical replicates. qRT-PCR data in A represent the *MAP7D1/ACTB* values obtained by normalizing *MAP7D1* mRNA expression levels in each experimental group to its respective *ACTB* mRNA expression level using the ΔΔCT method and compared with those of the control experiment group. (B) Quantification of endogenous MAP7D1 protein levels in control and patient fibroblasts. The expression level of endogenous MAP7D1 protein was analyzed by western blotting (left) with β-actin as an internal loading control. The experiment was performed in three biological replicates. Quantification of band densities is represented in the bar graph (right). Data were normalized to those of β-actin and compared with those of the control experiment group. (C) Confocal images showing colocalization of MAP7D1 protein with microtubules in control and patient fibroblasts (left and right panel, respectively) using immunocytochemistry. Antibody labeling for MAP7D1 is shown red, microtubules (stained for β-tubulin) are shown in green. Shown are representative of three biological replicates including two independent technical replicates. Merged images are shown on the right of each panel. Scale bars: 20 µm. (D) Quantification of the cell area in control and patient fibroblasts is represented in the bar graph (*n*≥45). (E) Quantification of the fluorescence intensity [measured as active fluorescent unit (AFU)] of microtubules in control and patient fibroblasts is represented in the bar graph (*n*≥50). *t*-test was used for all statistical analysis. Error bars represent the mean±s.e.m. *P<0.05, ***P<0.001, ****P<0.0001.

A previous study conducted with an SDS cohort discovered a novel, possibly causative variant in the MAP7D1, i.e. the MAP7D1:p.R201W variant, in a Turkish patient (Oguz, 2020). To analyze the causality of this variant with the disease, we first investigated whether the MAP7D1:p.R201W mutation causes expressional changes in mRNA and protein levels. Real-time PCR and immunoblotting analyses were performed in control and patient fibroblasts, and the results indicated that both mRNA (Fig. 1A) and protein (Fig. 1B) levels of MAP7D1 were significantly lower in SDS patient fibroblasts than in control fibroblasts. We also examined colocalization of MAP7D1 with microtubules, since the MAP7D1:p.R201W mutation resides in the microtubule-binding domain of MAP7D1 and is likely to impair microtubule−MAP7D1 interaction. Immunocytochemistry showed that MAP7D1 colocalized with microtubules in control fibroblasts, and microtubules exhibited stable structures as bundled filaments (Fig. 1C, left panels). By contrast, MAP7D1 harboring the p.R201W mutation in patient fibroblasts exhibited reduced colocalization with microtubules, resulting in the loss of stable microtubule structures (Fig. 1C, right panels) and a reduction in the cell size (Fig. 1D). Moreover, when the fluorescence intensity of microtubules was measured to quantitatively compare the intensity alterations observed between control and patient fibroblasts, microtubule densities were found to be significantly decreased in patient fibroblasts compared to control fibroblasts (Fig. 1E). These results suggest that the MAP7D1:p.R201W mutation in the microtubule-binding domain of MAP7D1 impairs its association with and stabilization of microtubules.

**Fig. 2.**
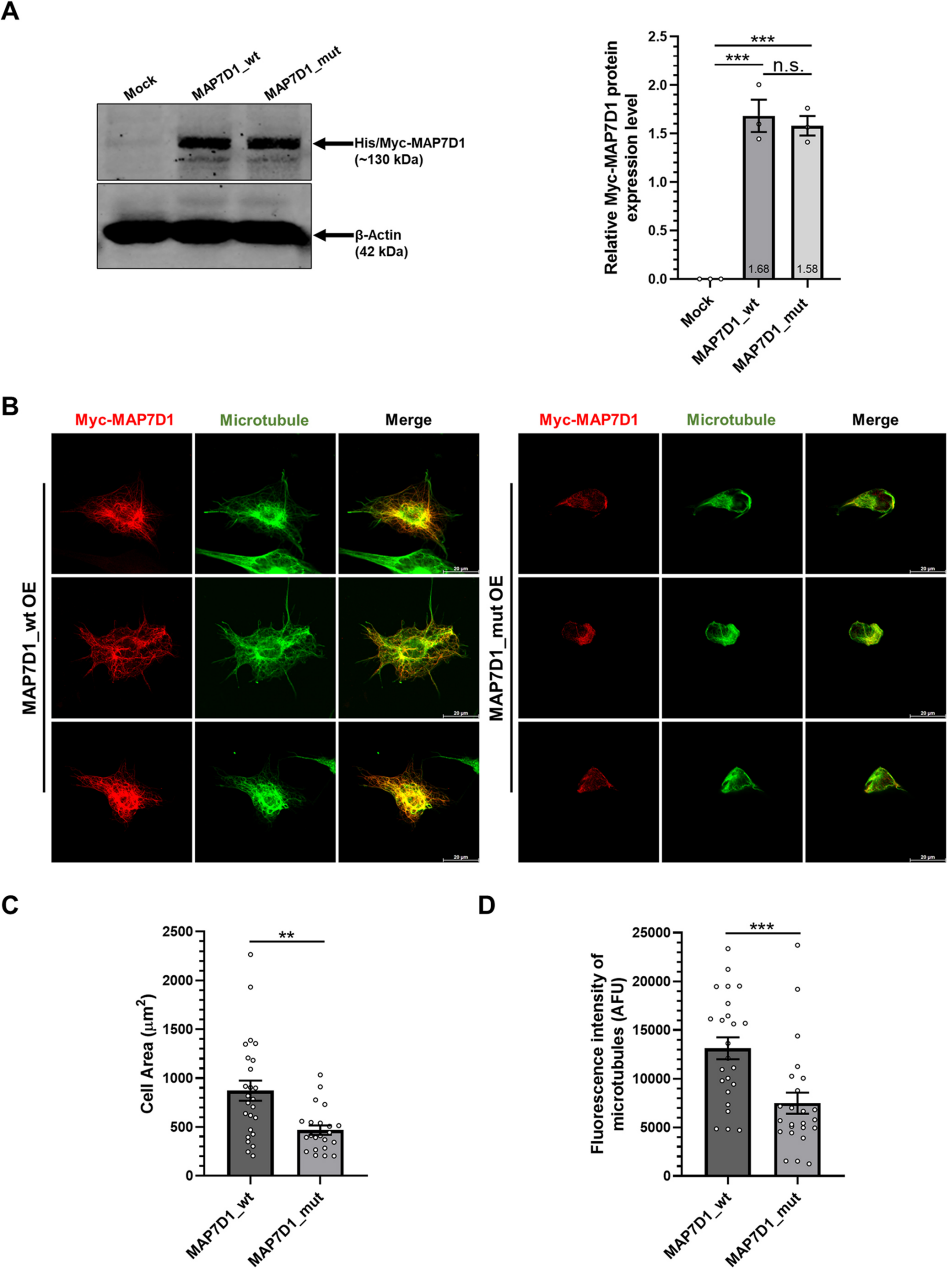
**Colocalization of MAP7D1 protein with microtubules decreases with the MAP7D1:p.R201W mutation.** (A) Western blotting (left) for MAP7D1 protein levels in T98G cells that had been transfected for 24 h with constructs expressing wild-type (MAP7D1_wt) or mutant (MAP7D1_mut) MAP7D1 or with a control construct (mock). Levels of overexpressed (OE) MAP7D1 protein were analyzed by immunoblotting with Myc-tag antibody (for His/Myc-MAP7D1 protein). Expression levels of β-actin were used as internal loading control. The experiment was performed in three biological replicates. Quantification of band densities is represented in the bar graph (right). (B) Confocal images showing colocalization of overexpressed MAP7D1 with microtubules in T98G cells by immunocytochemistry. Cells immunolabeled with Myc-tag antibody against exogenous MAP7D1 are shown in red, microtubules (stained for β-tubulin) are shown in green. Images are representative of two biological replicates, each including two independent technical replicates. Scale bars: 20 µm. (C) Quantification of the cell area of T98G cells overexpressing wild-type or mutant MAP7D1 protein is represented in the bar graph (*n*≥20). (D) Quantification of the fluorescence intensity of microtubules is represented in the bar graph (*n*≥25). One-way ANOVA (A) or *t*-test (C,D) was used for statistical analysis. Error bars represent the mean±s.e.m. **P<0.01, ***P<0.001. n.s., not significant (*P*>0.05).

To investigate whether the decrease in the microtubule density observed in patient fibroblasts was due to the direct effect of the MAP7D1:p.R201W mutation, and independent from the patient cells, wild-type and mutant MAP7D1-expressing constructs were generated. For this, wild-type and mutant MAP7D1 proteins were overexpressed in T98G glioblastoma cells, and we found no significant difference in overexpression levels of these proteins due to the mutation (Fig. 2A). Upon this, immunocytochemistry analysis was performed in T98G cells overexpressing either wild-type or mutant MAP7D1 protein, to determine the effect of the MAP7D1:p.R201W mutation on microtubule density. Immunocytochemistry analysis of the T98G cells showed results similar to those of fibroblasts (Fig. 1C), i.e. wild-type MAP7D1 protein colocalized with microtubules, resulting in dense and filamentous microtubule structures (Fig. 2B, left panels), whereas colocalization of mutant MAP7D1 protein with microtubules was diminished, leading to a decrease in microtubule stabilization and, hence, filamentous microtubule structures (Fig. 2B, right panels), consequently resulting in decreased cell size (Fig. 2C). In the following, fluorescence intensity of microtubule mass was quantitatively analyzed depending on the MAP7D1:p.R201W mutation, and we found that the density of microtubules was reduced in T98G cells overexpressing the mutant MAP7D1 protein (Fig. 2D). These findings indicated that the p.R201W mutation, situated in the microtubule-interaction domain of MAP7D1, causes a decrease in microtubule intensity, resulting in microtubule destabilization. These results confirm that the effect this mutation has on microtubules is independent to its effect on decreased MAP7D1 protein levels in patient fibroblasts.

**Fig. 3.**
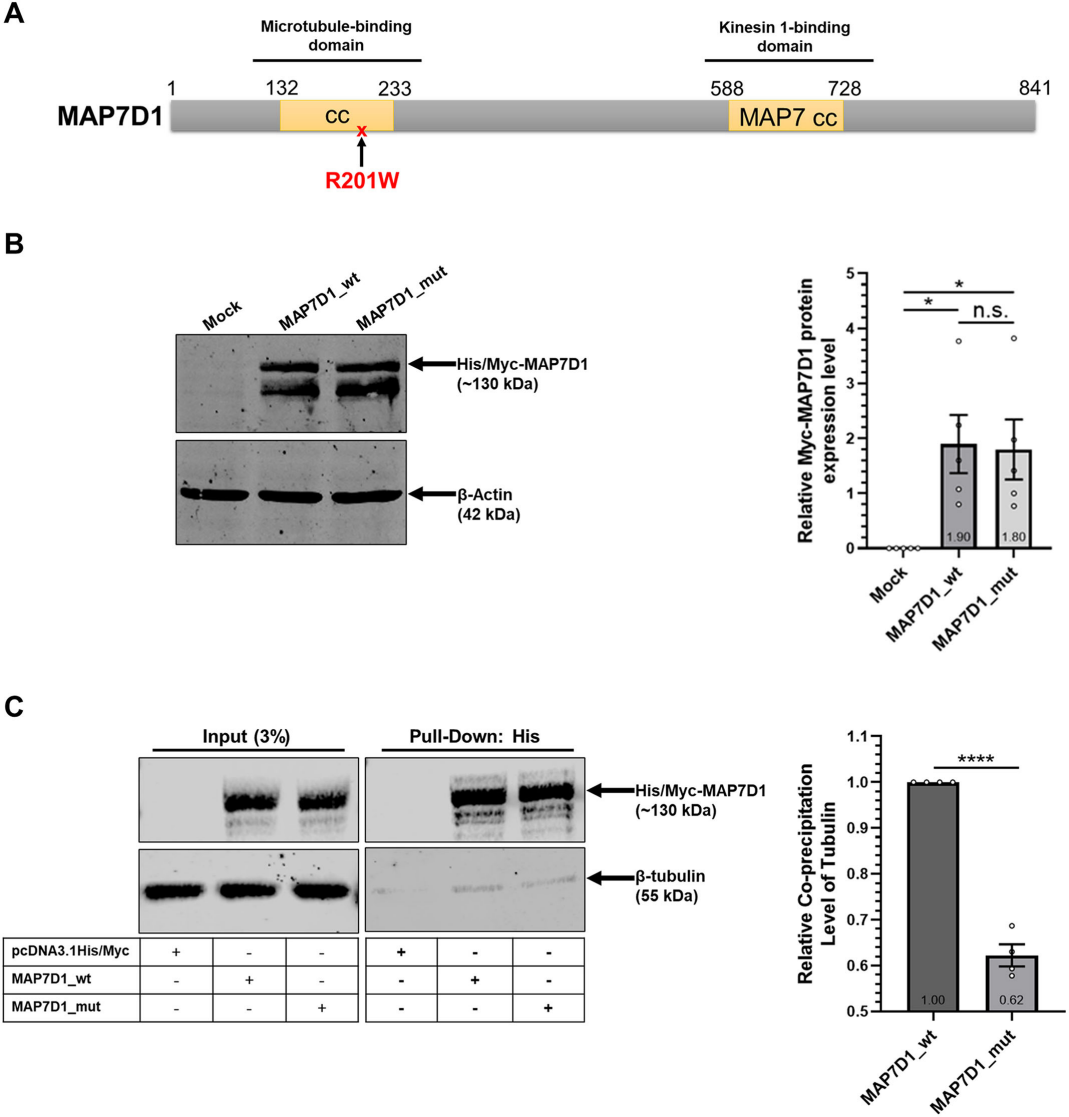
**The interaction between microtubules and MAP7D1 is impaired by the MAP7D1:p.R201W mutation within the microtubule-binding domain of the MAP7D1 protein.** (A) Schematic of the microtubule- and kinesin 1-binding domains in MAP7D1, and the site damaged by the p.R201W mutation. (B) Western blotting (left) for MAP7D1protein levels in HEK293T cells that had been transfected for 24 h with constructs expressing wild-type (MAP7D1_wt) or mutant (MAP7D1_mut) MAP7D1, or with a control construct (mock). Levels of overexpressed (OE) MAP7D1 protein were analyzed by immunoblotting with Myc-tag antibody (for His/Myc-MAP7D1 protein). Expression levels of β-actin were used as internal loading control. The experiment was performed in five biological replicates. Quantification of band densities is represented in the bar graph (right). (C) Analysis of interaction between microtubules and MAP7D1_wt or MAP7D1_mut overexpressed in HEK293T cells. MAP7D1 proteins were purified and precipitated from cell lysates (see Materials and Methods). Left: Co-precipitation of MAP7D1_wt or MAP7D1_mut with β-tubulin was assessed by western blotting using Myc-tag (for His/Myc-MAP7D1 protein) and β-tubulin antibodies. The experiment was performed in three biological replicates. Right: Quantification of co-precipitation of β-tubulin with MAP7D1_wt or MAP7D1_mut. Data were normalized to precipitated Myc-MAP7D1 levels and the MAP7D1_wt experiment group. One-way ANOVA (B) or *t*-test (C) were used for statistical analysis. Error bars represent the mean±s.e.m. *P<0.05, ****P<0.0001. n.s., not significant (*P*>0.05).

The study by Oguz, identifying the MAP7D1:p.R201W variant, *in silico* analysis showed that this variant alters the 3D structure of MAP7D1, especially of its microtubule-binding site (Oguz, 2020). To elucidate whether the reduction in microtubule stabilization was the direct effect of the mutation within the microtubule-binding domain (Fig. 3A), we investigated the physical interaction between microtubules and MAP7D1 depending on the p.R201W mutation. We transfected HEK293T cells with plasmid constructs expressing wild-type or mutant *MAP7D1* and analyzed respective expression levels by western blotting. Wild-type and mutant MAP7D1 proteins were overexpressed at similar levels (Fig. 3B). The pull-down analysis using HEK293T cells overexpressing wild-type or mutant MAP7D1 showed that tubulin co-precipitated with wild-type and mutant MAP7D1; however, co-precipitation of tubulin with the mutant MAP7D1 protein was significantly decreased by ∼40% relative to that with wild-type MAP7D1 protein (Fig. 3C). This result indicates that the p.Arg201Trp mutation – owing to changes to the 3D structure of MAP7D1 – greatly disrupts the physical interaction between microtubules and MAP7D1. All these findings demonstrate that the p.R201W mutation in the microtubule-binding domain of MAP7D1 drastically inhibits binding of MAP7D1 to microtubules, leading to unstable microtubules and reduced cell size.

### The MAP7D1:p.R201W mutation causes aberrant mitosis by inducing mitotic spindle instability in SDS patient cells

**Fig. 4.**
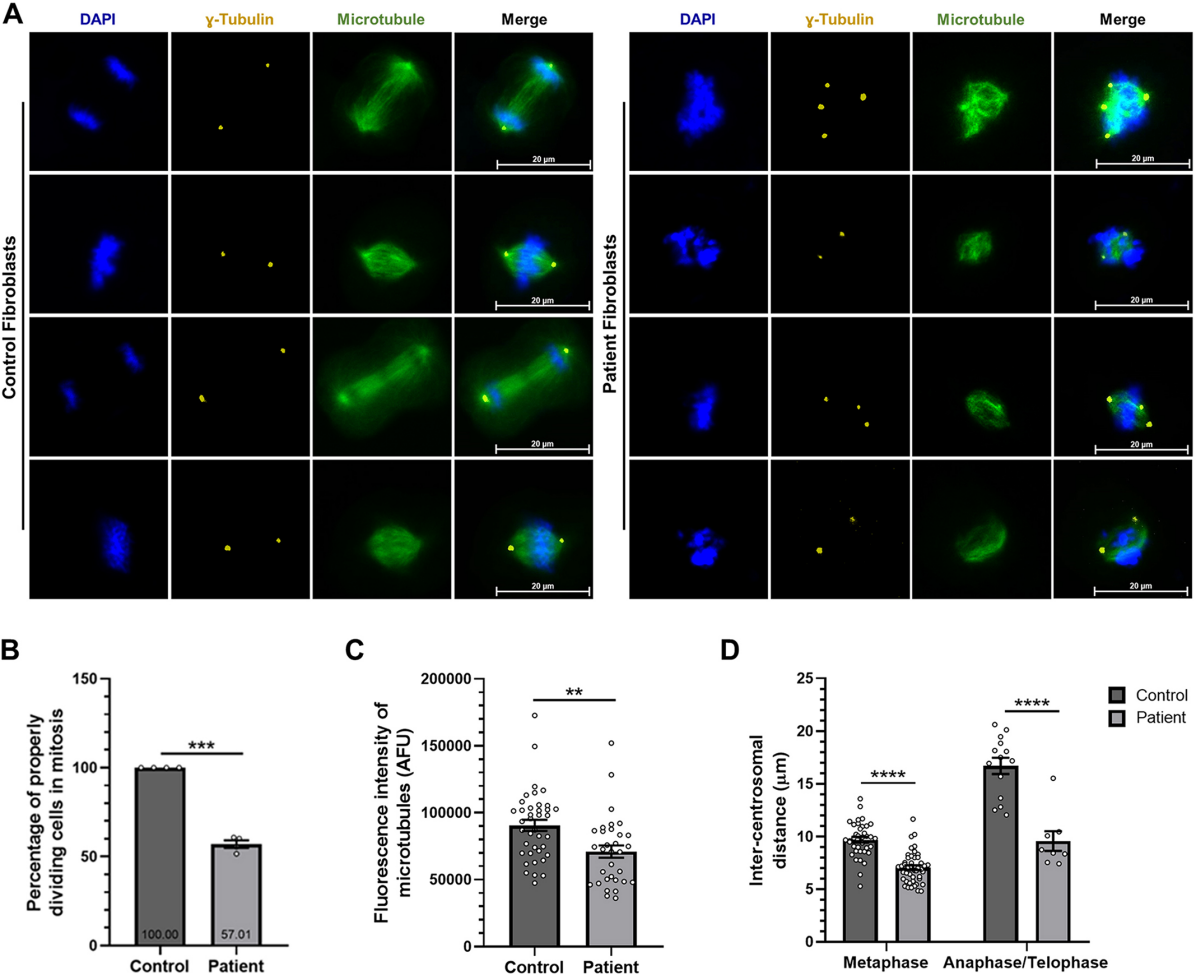
**Mitotic abnormalities develop in SDS patient cells.** (A) Analysis of mitotic spindle stability in control and SDS patient fibroblasts. Cells were synchronized and fixed (see Materials and Methods), followed by immunocytochemistry. Centrosomes were stained against γ-tubulin (yellow) and microtubules against β-tubulin (green). Nuclei (chromosomes) were stained with DAPI (blue). Shown are representative fluorescence images of three biological replicates including three independent technical replicates. Scale bars: 20 µm. (B) The percentage of normal mitotic cells in control and patient cells is represented in the bar graph (*n*≥400). Data were normalized to the control experiment group. (C) Quantification of fluorescence intensity of mitotic microtubules in control and patient cells (*n*≥30). (D) Quantification of the distance between two centrosomes in control and patient cells at metaphase and anaphase/telophase (*n*=30). *t*-test (B,C) or two-way ANOVA (D) was used for all statistical analysis. Error bars represent the mean±s.e.m. **P<0.01, ***P<0.001, ****P<0.0001.

Previous studies revealed that mitotic spindle instability and mitotic abnormalities can arise in SDS patient cells, which may cause bone marrow failure and leukemogenesis (Austin et al., 2008; Sera et al., 2024). Therefore, we initially examined whether any mitotic abnormalities develop in SDS patient cells carrying the MAP7D1:p.R201W mutation. Control and patient fibroblasts were synchronized at S phase of the cell cycle by using the thymidine block and fixed every 30 min during mitosis for immunocytochemistry analysis. Control fibroblasts had stable mitotic spindle structures between two centrosomes, with chromosomes properly positioned on the spindle (Fig. 4A, left panels). By contrast, patient fibroblasts had irregular mitotic spindles between two or more centrosomes, with chromosomes unable to align, lagging behind and remaining unevenly distributed (Fig. 4A, right panels). Furthermore, the percentage of patient fibroblasts exhibiting mitotic anomalies was quantified relative to control fibroblasts, showing that ∼40% of patient fibroblasts were dividing abnormally (Fig. 4B). Moreover, fluorescence intensity of microtubules and inter-centrosomal distance were quantified in both control and patient fibroblasts. The results showed a significant reduction in mitotic microtubule density in the patient fibroblasts (Fig. 4C), and a significant decrease in the distance between two centrosomes during metaphase and anaphase/telophase (Fig. 4D) within patient fibroblasts containing bipolar spindle structures. These findings indicate that patient cells display abnormal mitotic spindle assembly and exhibit aberrant cell division.

**Fig. 5.**
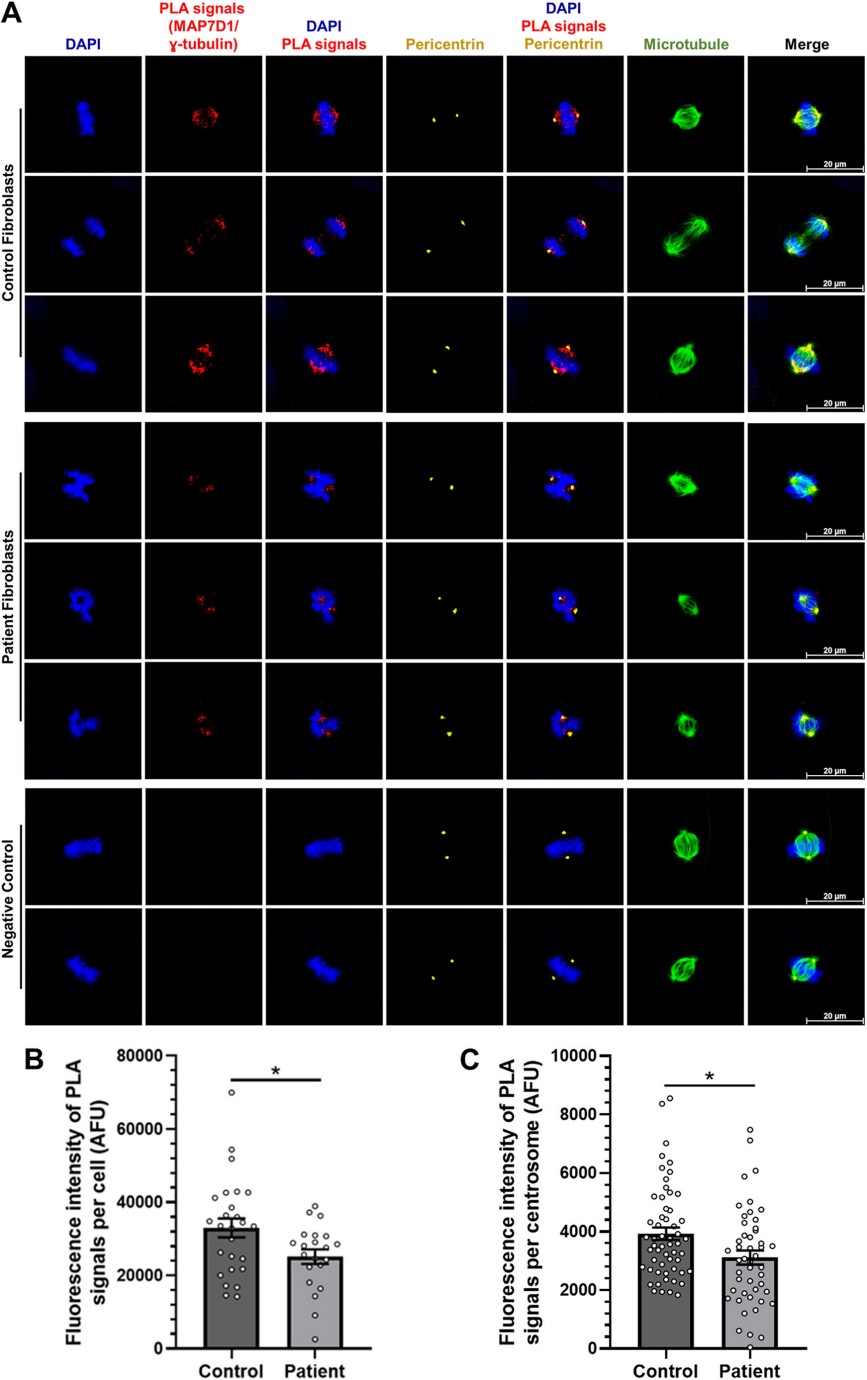
**The association of MAP7D1 with centrosomes is reduced in the presence of the MAP7D1:p.R201W mutation.** (A) PLA analysis between the MAP7D1 and γ-tubulin in the control and SDS patient fibroblasts. Cells were synchronized using double thymidine block and fixed with methanol. For PLA analysis, cells were immunocytochemically labeled with MAP7D1 antibody for endogenous MAP7D1 and γ-tubulin antibody for centrosome. PLA signals indicating the association between MAP7D1 and γ-tubulin are shown in red. Cells were either labeled with anti-pericentrin antibody to stain centrosomes (yellow) to determine whether PLA signals are in specific regions or anti-β-tubulin antibody to stain for microtubules (green). Nuclei chromosomes were stained with DAPI (blue). Negative controls comprised only γ-tubulin (upper panel) or only MAP7D1 (lower panel) primary antibody in the reaction of control fibroblasts to assess background signal and to ensure the specificity of the assay by confirming that PLA signals arise only from true protein-protein proximity. Images were captured by confocal microscope with a 63× objective and shown are representative of two biological replicates including three independent technical replicates. Scale bars: 20 µm. (B,C) Quantification of fluorescence intensity of PLA signals per cell (*n*≥20) (B) and per centrosome (*n*≥45) (C) in control and patient cells, *t*-test was used for statistical analysis. Error bars represent the mean±s.e.m. *P<0.05.

After detecting mitotic spindle abnormalities, multipolar centrosomes and shortening of the centrosome distance within SDS patient cells carrying the MAP7D1:p.R201W mutation, we comparatively examined whether there was a difference in interaction between wild-type and mutant MAP7D1 proteins with centrosomes, the microtubule organizing center (MTOC) of the cell. Proximity ligation assay (PLA) was performed with control and patient fibroblasts expressing endogenous MAP7D1 protein and the centrosomal component γ-tubulin. Specific PLA signals indicating the endogenous MAP7D1 protein association with γ-tubulin were observed in both control and patient fibroblasts at pericentrin regions, a part of the MTOC (Fig. 5A). Next, the intensity of PLA signals was analyzed to determine if there was a difference in the quantity of the interaction between the wild-type or mutant MAP7D1 protein and γ-tubulin relative to each other. The PLA signal intensity per cell (Fig. 5B) and centrosome (Fig. 5C) was significantly reduced in the patient fibroblasts compared to the control fibroblasts by ∼20-30% (Fig. 5B,C). These results reveal that the MAP7D1 protein interacts with the MTOC and that the presence of the MAP7D1:p.R201W mutation adversely affects this association.

**Fig. 6.**
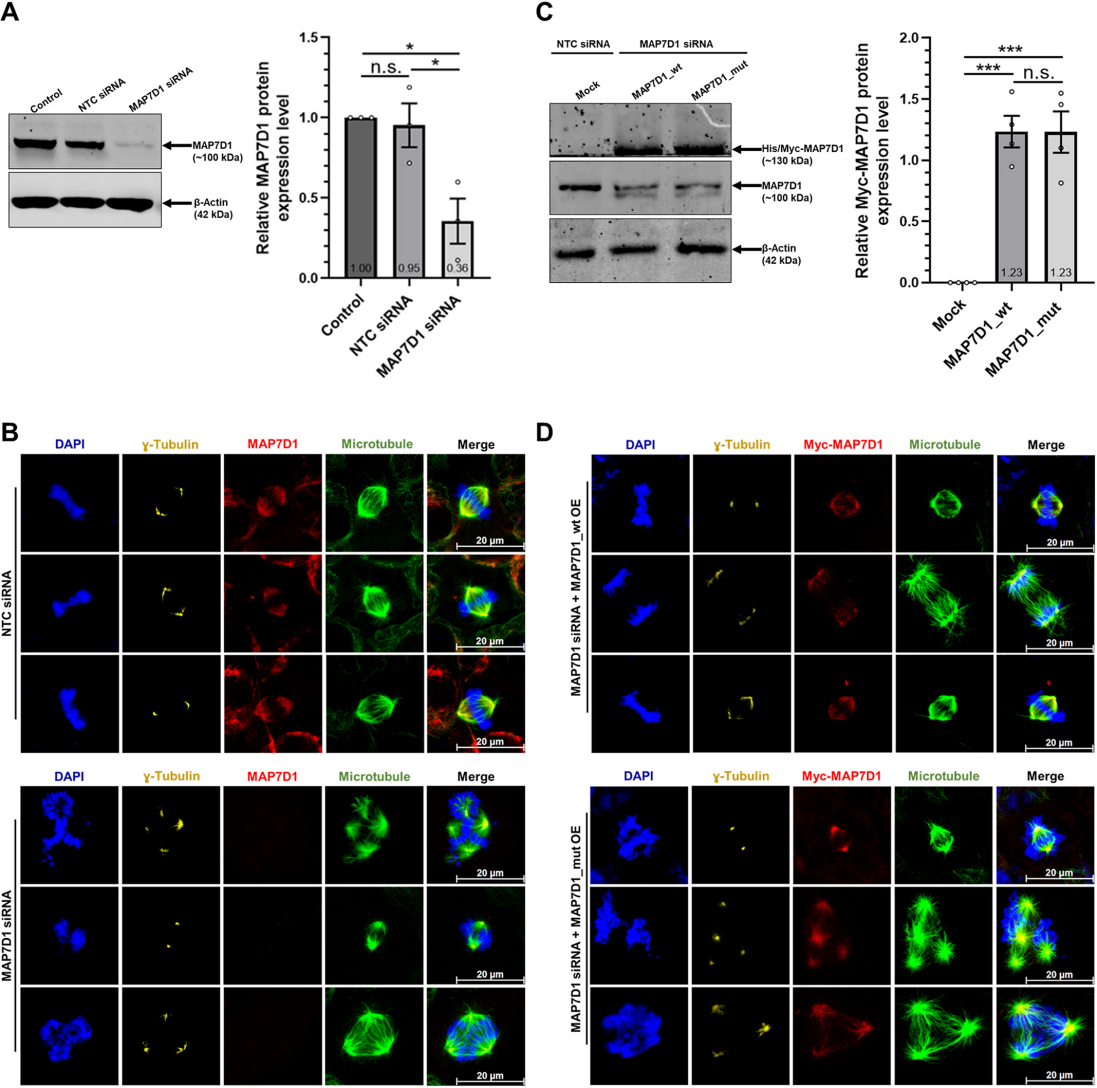
**MAP7D1 protein plays an essential role in mitosis.** (A-D) HEK293T cells were used to analyze the role of the human wild-type MAP7D1 protein (A,B) and mutant MAP7D1:p.R201W protein (C,D). For this, endogenous *MAP7D1* gene expression was silenced by using *MAP7D1*-targeting or non-targeting control (NTC) siRNA (MAP7D1 siRNA or NTC siRNA, respectively) for the first experimental set-up (panels A and B). In a second experimental set-up, endogenous *MAP7D1* gene expression in cells was silenced but then followed by transfection with plasmid constructs overexpressing either human wild-type MAP7D1 protein (MAP7D1_wt), mutant MAP7D1 protein (MAP7D1_mut) (panels C and D) or a control plasmid (mock). Protein levels of endogenous MAP7D1 (A) or exogenously expressed MAP7D1_wt or MAP7D1_mut (C) were analyzed by western blotting. β-actin was used as an internal control. Experiments were performed in three biological replicates. Quantification of protein expression is shown in the bar graphs on the right of each panel. Data were normalized to β-actin and/or control experiment groups. One-way ANOVA was used for statistical analysis. Error bars represent the mean±s.e.m. *P<0.05, ***P<0.001. n.s., not significant (*P*>0.05). (B,D) Representative confocal images of synchronized HEK293T cells as described above, showing the effect of MAP7D1 proteins on mitosis. HEK293T cells expressing NTC siRNA or MAP7D1 siRNA are shown in B. HEK293T cells dually expressing MAP7D1 siRNA and either MAP7D1_wt or MAP7D1_mut constructs are shown in D. Immunostaining for MAP7D1 is shown in red, centrosomes were stained for γ-tubulin (yellow), and microtubules were stained for β-tubulin (green). Nuclei (chromosomes) were stained with DAPI (blue). Images are representative of three biological replicates including two independent technical replicates. Scale bars: 20 µm.

In the genetic study identifying the pathogenic MAP7D1:p.R201W variant as a candidate variant, the patient also had several non-candidate variants (Oguz, 2020). Therefore, whether the mitotic abnormalities detected in SDS patient cells were driven directly by the mutation in the MAP7D1 protein was investigated. To do so, initially the role of endogenous MAP7D1 protein in mitosis was evaluated. For this purpose, expression of endogenous *MAP7D1* was silenced using siRNA in HEK293T cells (Fig. 6A) and immunocytochemistry analysis was performed following the synchronization of cells by using the double thymidine block method. In the presence of the MAP7D1 protein, HEK293T cells divided by establishing a stable mitotic spindle between two centrosomes (Fig. 6B, upper panels), whereas in the absence of MAP7D1, the cells divided by assembling multipolar or bipolar unstable mitotic spindles (Fig. 6B, lower panels). This result demonstrates that the cells divide abnormally in mitosis upon MAP7D1 depletion. This finding also suggests that MAP7D1 is necessary for cells to divide properly, implying that MAP7D1 is essential for the alignment of chromosomes and healthy progression of mitosis. To comparatively investigate the direct effects of the MAP7D1:p.R201W mutation on mitosis, HEK293T cells overexpressing wild-type or mutant MAP7D1 protein were analyzed by immunocytochemistry. Since SDS is an autosomal recessive disorder, endogenous *MAP7D1* expression was silenced using siRNA before overexpressing either the wild-type or the mutant MAP7D1 version in HEK293T cells (Fig. 6C), to account for the potential compensatory effect of endogenously expressed wild-type MAP7D1 protein on the overexpressed mutant version of MAP7D1 and, thus, to better observe the effect of the mutation on mitosis. Multipolar unstable mitotic spindle structures detected in MAP7D1-depleted cells disappeared and the cells regained their regular mitotic spindle structure when wild-type MAP7D1 protein was overexpressed (Fig. 6D, upper panels). However, when mutant MAP7D1 protein was overexpressed, cells divided improperly with unstable mitotic spindles between two or more centrosomes (Fig. 6D, lower panels), as observed in the absence of MAP7D1 (Fig. 6B, lower panels), implying that MAP7D1:p.R201W mutation may have a loss-of-function effect. Taken together, we revealed that the mitotic irregularities discovered in the SDS patient fibroblasts are directly caused by the MAP7D1:p.R201W mutation.

### MAP7D1:p.R201W mutation triggers accumulation of RPS14 in SDS patient cells

**Fig. 7.**
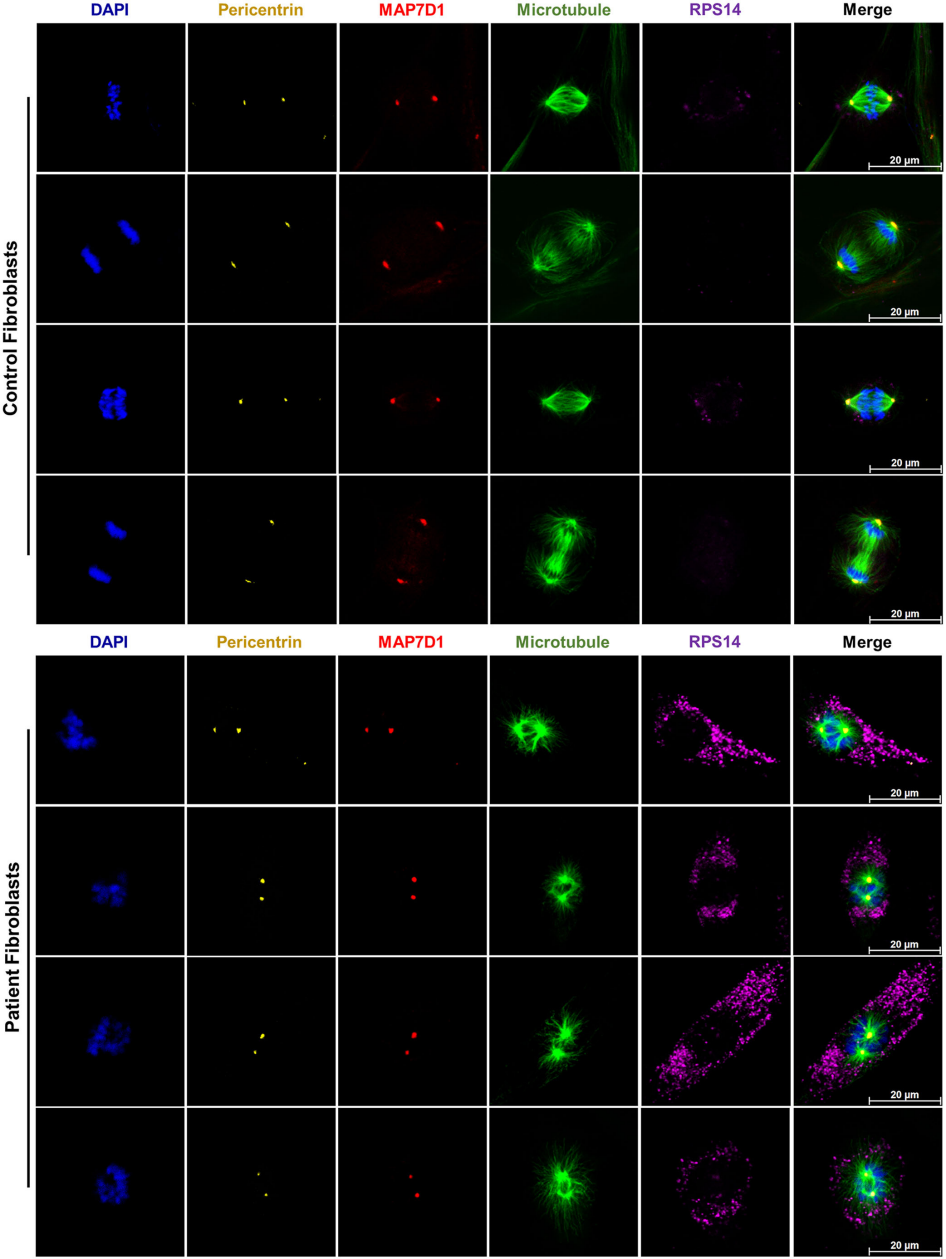
**RPS14 protein accumulates in the SDS patient cells.** Representative confocal images of synchronized control and SDS patient fibroblasts (top and bottom panels, respectively). RPS14 accumulation is observed in SDS patient cells exhibiting abnormal mitosis compared to properly dividing control cells. Immunostaining was for MAP7D1 (red) and RPS14 (purple). Centrosomes were stained for pericentrin (yellow), microtubules were stained for β-tubulin (green). Nuclei (chromosomes) were stained with DAPI (blue). Images are representative of two biological replicates, each including three independent technical replicates. Scale bars: 20 µm.

Since this study aimed to elucidate whether *MAP7D1* is a new gene related to SDS (generally is known as a ribosomopathy), we suggested that – if *MAP7D1* is a novel causative gene for SDS – it may also affect the function of ribosomal proteins, which are crucial to the etiology of SDS. Therefore, the connection between MAP7 family proteins as well as ribosomal proteins were examined by using the STRING database (RRID:SCR_005223), a biological database of known and/or predicted protein−protein interactions. We found that MAP7, a paralog of MAP7D1, may interact with RPS14 (Fig. S1). The latter is encoded by the *RPS14* gene, a component of the small subunit of the ribosome (40S), and plays a role in the processing of 18S pre-ribosomal RNA (Boultwood et al., 2012). Then, the association between MAP7D1 and RPS14 was compared in control and patient fibroblasts by immunocytochemistry analysis. It was observed that MAP7D1 predominantly localized to the centrosome in both control and patient fibroblasts, and did not colocalize with RPS14 (Fig. 7). Additionally, RPS14 was found to accumulate more densely in improperly dividing patient fibroblasts as opposed to normally dividing control fibroblasts (Fig. 7). These findings implied that, in SDS patient cells, mutant MAP7D1 protein influences accumulation of RPS14, even if it does not directly colocalize with RPS14.

**Fig. 8.**
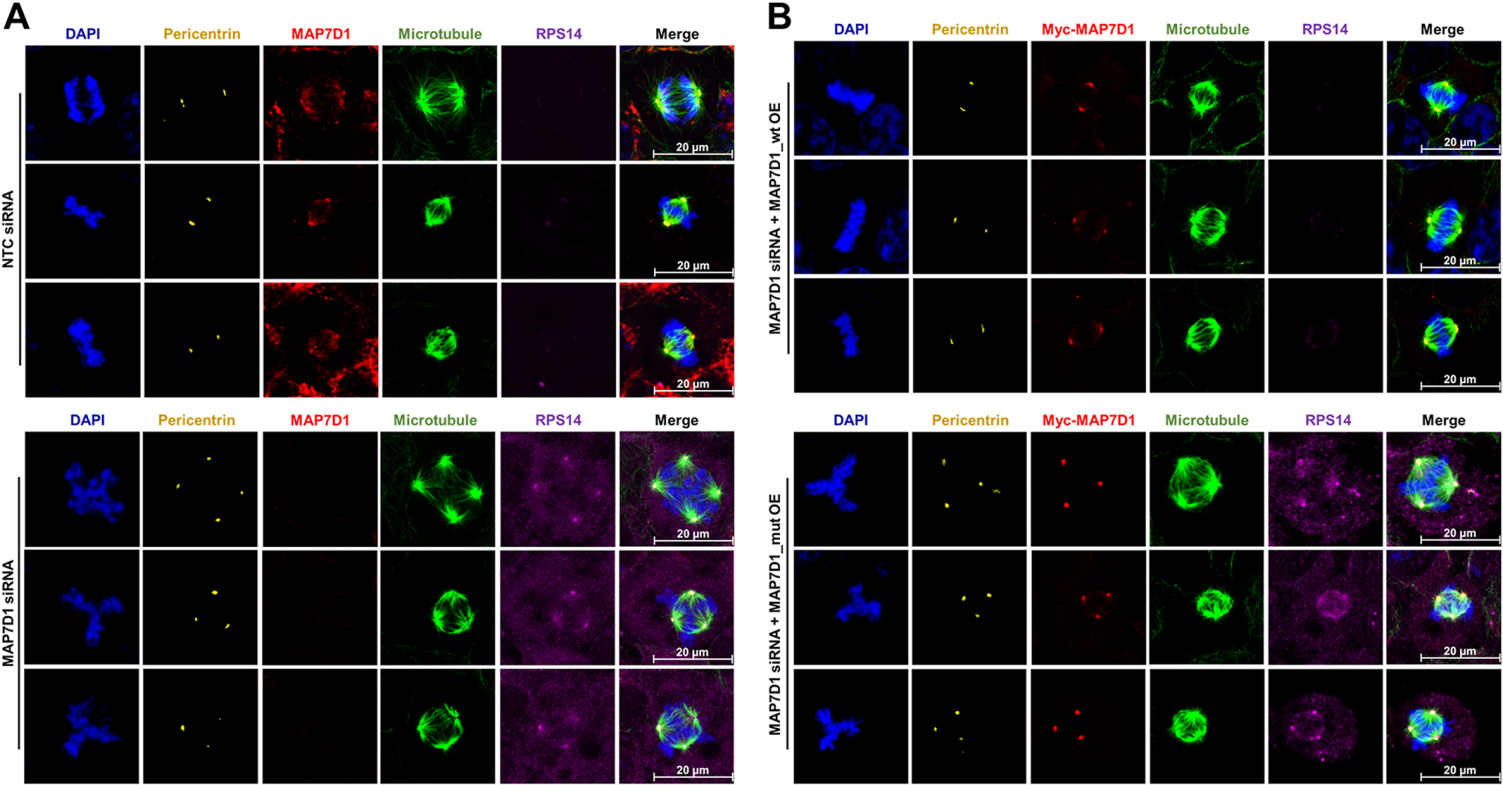
**The MAP7D1:p.R201W mutation directly leads to accumulation of RPS14.** Analysis of the effect MAP7D1 mutation has on RPS14 protein levels following the same experimental set-up as described for Fig. 6. (A) Representative confocal images of synchronized HEK293T cells expressing non-targeting control siRNA (NTC siRNA; top panel) or *MAP7D1*-targeting siRNA (MAP7D1 siRNA; bottom panel) to suppress expression of endogenous MAP7D1 protein. Accumulation of RPS14 is observed in HEK293T cells that express MAP7D1 siRNA and, thus, divide abnormally. (B) Representative confocal images of synchronized HEK293T cells dually expressing MAP7D1 siRNA and either human wild-type MAP7D1 protein (MAP7D1_wt OE; top panel) or the mutant MAP7D1 protein (MAP7D1_mut; bottom panel). Accumulation of RPS14 is observed in HEK293T cells that dually express MAP7D1 siRNA with MAP7D1_mut construct and, thus, divide abnormally. Immunostaining for MAP7D1 is shown in red, centrosomes were stained for pericentrin (yellow), and microtubules were stained for β-tubulin (green). Nuclei (chromosomes) were stained with DAPI (blue). Images are representative of two biological replicates, each including two independent technical replicates. Scale bars: 20 µm.

To investigate whether RPS14 accumulation observed in abnormally dividing SDS cells is a direct effect of the MAP7D1:p.R201W mutation, immunocytochemistry analysis was performed in HEK293T cells that had been cell cycle synchronized in the presence or absence of MAP7D1 (Fig. 8). While no RPS14 accumulation was observed in properly dividing HEK293T cells in the presence of MAP7D1 (Fig. 8A, upper panels), RPS14 accumulation was observed in abnormally dividing HEK293T cells when *MAP7D1* had been silenced with siRNA (Fig. 8A, lower panels), showing that MAP7D1 may have a role in the regulation of RPS14, a ribosomal protein. Following this, we investigated whether the MAP7D1:p.R201W mutation itself leads to RPS14 accumulation. As previously explained for the experimental set-up described for Fig. 6, the effect of the MAP7D1:p.R201W expression in regard to accumulation of RSP14 protein was examined. This was done by suppressing endogenous *MAP7D1* expression in HEK293T cells and then overexpressing human wild-type *MAP7D1* or the MAP7D1:p.R201W mutant. As a result, HEK293T cells overexpressing mutant MAP7D1 (MAP7D_mut OE) and, thus, dividing improperly, showed extensive accumulation of RPS14, as opposed to properly dividing cells overexpressing wild-type MAP7D1 (MAP7D_wt OE) (Fig. 8B). Therefore, RPS14 accumulation in improperly dividing cells that lack MAP7D1, as well as in cells in which endogenous expression of MAP7D1 had been silenced but that overexpress mutant MAP7D1, suggest that this effect is due to a loss-of-function mutation. This finding also indicates that the RPS14 accumulation in SDS fibroblasts was caused by the MAP7D1:p.R201W mutation. All these findings together suggest that MAP7D1, like SBDS, may play a dual role in the cell by influencing ribosomal proteins in addition to its microtubule-associated function, and that the MAP7D1:p.R201W mutation may contribute to SDS by disrupting these two cellular processes in the etiology of SDS.

## DISCUSSION

SDS is characterized by bone marrow failure and inherited in autosomal recessive manner (Shwachman et al., 1964; Dror et al., 2011; Nelson and Myers, 2018). Previous studies on SDS have associated it with mutations within genes that are involved in ribosome biogenesis and, hence, the disease is classified as a ribosomopathy (Narla and Ebert, 2010; Nelson and Myers, 2018; Warren, 2018). However, recent studies have revealed that the SBDS protein, responsible for >90% of SDS cases, has microtubule-associated roles alongside its ribosome-related functions (Austin et al., 2008; Orelio et al., 2009). Thus, while these studies imply that microtubule-associated cellular processes are important for SDS, no study has so far investigated the role of microtubule-associated proteins in the etiology of SDS.

In this study, we, for the first time, investigated the causal connection between SDS and the microtubule-associated MAP7D1:p.R201W mutation, and found it to reduce the transcriptional and translational expression levels of MAP7D1 (Fig. 1A,B). Also, by using *in situ* analyses we observed that colocalization of mutant MAP7D1 protein with microtubules was reduced in patient fibroblasts, decreasing microtubule density and cell size (Fig. 1C-E). However, it was unclear whether the underlying reason for these microtubule-related alterations in patient fibroblasts was due to decreased MAP7D1 protein levels caused by the mutation or due to the specific effect of the MAP7D1:p.R201W mutation on microtubules. To clarify this, we created recombinant wild-type and mutant MAP7D1 constructs that expressed both protein at equal levels (Fig. 2A). Immunocytochemistry analysis of T98G cells overexpressing these constructs showed that the microtubule density, and, hence, the cell sizes were reduced in the presence of the mutant MAP7D1 (Fig. 2B-D). Consequently, even if wild-type and mutant MAP7D1 proteins were expressed at equal levels, the observed reduction in microtubule density and cell size was similar to those observed with the patient fibroblasts; hence, the effect on microtubules is directly attributable to the MAP7D1 mutation specifically. Moreover, the physical interaction between microtubule and MAP7D1 was significantly impaired in the presence of the MAP7D1:p.R201W mutation (Fig. 3C). Together, these results revealed that the MAP7D1:p.R201W mutation weakens interaction of MAP7D1 with microtubules due to the mutation residing in the microtubule-binding site, resulting in a decrease in intracellular microtubule density and, thus, a decrease in cell size, which is independent to its effect on MAP7D1 protein expression.

The SBDS protein, associated with >90% of SDS cases, has been shown to stabilize microtubules by binding to them and by localizing to mitotic spindles (Austin et al., 2008; Orelio et al., 2009). In one study, primary bone marrow stromal cells of SDS patients with *SBDS* mutations have been shown to divide improperly, producing multipolar spindles between more than two centrosomes during mitosis (Austin et al., 2008). Furthermore, treatment of SDS lymphoblast cells with the microtubule-destabilizing agent nocodazole or the microtubule-stabilizing agent taxol results in mitotic arrest or resistance to mitotic delay, respectively, suggesting that mitotic spindle instability in SDS may play a role in the development of bone marrow failure and leukemogenesis (Austin et al., 2008). Moreover, a recent study revealed that the myeloblastic 32Dcl3 cell model of SDS, harboring mutations in the *SBDS* gene, exhibited abnormally accelerated mitosis and heightened chromosomal instability, with lagging chromosomes observed in almost 50% of these cells (Sera et al., 2024). In this study, we showed that 40% of SDS cells carrying the MAP7D1:p.R201W mutation, resulting in reduced microtubule−MAP7D1 interaction, exhibited abnormal cell division, forming bipolar or multipolar mitotic spindles during mitosis (Fig. 4A,B). Although SDS is inherited in an autosomal recessive manner, the presence of properly dividing cells in SDS fibroblasts suggests that another MAP7 family protein can compensate for the mutant MAP7D1 protein. Members of the MAP7 family are expressed at different levels in different cells and tissues. According to The Human Protein Atlas tool (RRID:SCR_006710), MAP7 and MAP7D1 are highly expressed in brain, skin tissues and immune system-related cells, such as lymphoid and bone marrow, MAP7D2 is expressed in brain and testis tissues, and MAP7D3 in male and female tissues. Therefore, MAP7 may functionally compensate for mutant MAP7D1 in SDS fibroblasts. Supporting this idea, Dullovi and colleagues previously described that, after application of gamma-irradiation to cause DNA damage in cells expressing different levels of MAP7 and MAP7D1, defects in DNA repair were observed only when both MAP7 and MAP7D1 were silenced together, but not when MAP7 or MAP7D1 was silenced separately, pointing out that these two proteins can functionally compensate for each other (Dullovi et al., 2023).

Also, we found that mitotic microtubule intensity was reduced in SDS fibroblasts, resulting in an unstable mitotic spindle with non-aligned lagging chromosomes (Fig. 4A,C). Moreover, the distance between two centrosomes in properly dividing control fibroblasts and in abnormally dividing patient fibroblasts with bipolar unstable mitotic spindle assembly was measured and, in this analysis, we excluded patient fibroblasts with multipolar unstable mitotic spindle assembly because it was unclear which two centrosomes to use for the measurement. As a result, the inter-centrosomal distance was found to be decreased during metaphase and anaphase/telophase in SDS fibroblasts (Fig. 4D). The interaction of wild-type or mutant MAP7D1 proteins with the centrosome was also examined by using PLA, and interaction of MAP7D1 with the centrosome was found to decrease due to the mutation (Fig. 5). Consequently, we demonstrated the occurrence of mitotic spindle instability and misaligned lagging chromosomes in SDS fibroblasts. Together with high expression level of MAP7D1 in bone marrow, these findings suggest that the mitotic aberrations seen in SDS patient fibroblasts carrying the MAP7D1:p.R201W mutation may contribute to bone marrow failure and leukemogenesis, similar to those seen in SDS patients that carry *SBDS* mutations.

Moreover, analysis of whole-exome sequencing and homozygosity mapping performed in a genetic study to investigate the SDS patient carrying the MAP7D1:p.R201W variant identified two more variants in that patient, i.e. of dipeptidyl peptidase 4 (*DPP4*) and of sodium voltage-gated channel alpha subunit 9 (*SCN9A*) – DPP4:c.1876A>G,p.I626V and SCN9A:c.2868delA,p.L956Ffs*2, respectively (Oguz, 2020). Mutations in *SCN9A*, which encodes a protein responsible for transmitting pain signals, are known to cause congenital insensitivity to pain, a disorder that impairs the ability to perceive physical pain (Nilsen et al., 2009; Staud et al., 2011; Klein et al., 2013; Zhou et al., 2013; Shorer et al., 2014). When SCN9A-related phenotypes were evaluated, it was determined that the loss-of-function mutation in *SCN9A* explained the insensitivity of the SDS patient to pain but not the bone marrow failure, pancreatic insufficiency, or neurological abnormalities [Online Mendelian Inheritance in Man (OMIM) 243000 and RRID:SCR_006437). The pathogenicity of the variant in *DPP4*, which encodes a serine exopeptidase that regulates physiological processes, such as cell differentiation, cell adhesion and apoptosis, as well as of a variant in microtubule-associated *MAP7D1*, were investigated by using PredictSNP (RRID:SCR_006327). Whereas the *DPP4* variant DPP4:c.1876A>G was neutral, the MAP7D1:c.601C>T, p.Arg201Trp variant was found to be deleterious. Considering these findings, it remained uncertain whether the mitotic abnormalities in SDS fibroblasts were directly attributable to the MAP7D1:p.R201W mutation, because the patient possessed two additional non-candidate variants, even if they were eliminated by genetic analyses, which differed from the healthy control. To further understand this, we used HEK293T cells to initially investigate the role of MAP7D1 in mitosis. Multipolar or bipolar unstable mitotic spindle assembly leading to abnormal cell division was observed upon MAP7D1 silencing (Fig. 6B), indicating that MAP7D1 is an essential protein for proper progression of mitosis. A recent study has shown that DNA-damaged cells are arrested in G1 phase of the cell cycle upon silencing of MAP7D1 that was found to interact with DNA damage repair proteins (Dullovi et al., 2023). In conjunction with that study, our findings suggest that silencing of MAP7D1 induced mitotic defects not only by interfering with DNA damage repair proteins but also by impairing mitotic spindle assembly and stability. Following determining the importance of MAP7D1 in mitosis, we researched the effect of the MAP7D1:p.R201W mutation during mitosis in HEK293T cells by overexpressing wild-type or mutant MAP7D1 proteins. To exclude the possibility that endogenous MAP7D1 in HEK293T cells might compensate for the effect of overexpressed mutant MAP7D1 and since SDS is an autosomal recessive disorder, wild-type or mutant MAP7D1 proteins were overexpressed after suppression of endogenous *MAP7D1* with siRNA. Overexpression of wild-type MAP7D1 protein rescued the abnormal division in HEK293T cells caused by depletion of MAP7D1 and the cells divided properly by forming a bipolar stable mitotic spindle. Meanwhile, mitotic abnormalities seen with overexpression of mutant MAP7D1 in HEK293T cells were similar to those after silencing of endogenous MAP7D1 (Fig. 6D). This finding indicates that the MAP7D1:p.R201W mutation is directly responsible for the abnormal division in SDS fibroblasts. Furthermore, the MAP7D1:p.R201W mutation appeared to be a loss-of-function mutation.

To date, SDS has been associated with defects in ribosome biogenesis and, therefore, is classified a ribosomopathy (Narla and Ebert, 2010; Warren, 2018). In recent years, with the discovery that the SBDS protein has a role in microtubule stabilization in addition to its ribosome biogenesis function and, thus, detection of mitotic abnormalities in SDS cells, microtubule-related processes have become important in the etiology of SDS but ribosome-related defects underlying the disease cannot be ignored. Therefore, we evaluated whether the recently identified MAP7D1:p.R201W mutation affects the regulation of ribosomal proteins. When searching the STRING database for proteins interacting with members of the MAP7 family, the MAP7D1 paralog MAP7 was shown to interact with RPS14, a component of the 40S ribosomal subunit (Boultwood et al., 2012). Therefore, we analyzed localization of RPS14 in SDS patient fibroblasts harboring the MAP7D1:p.R201W mutation and found that RPS14 accumulated in SDS patient fibroblasts with mitotic aberrations (Fig. 7). Furthermore, RPS14 did not colocalize with wild-type or mutant MAP7D1 protein but its accumulation was directly induced by the MAP7D1:p.R201W mutation (Figs 7 and 8). It is known that RPS14 accumulates in cells in response to nucleolar stress (Zhou et al., 2013; Lessard et al., 2018, 2019). To repair the damage caused by nucleolar stress, RPS14 inhibits proteins responsible for inactivation of the two main tumor suppressor proteins, cellular tumor antigen (p53) and retinoblastoma-associated protein (Rb), and activates these tumor suppressor proteins, thereby causing cell cycle arrest or cell death (Zhou et al., 2013; Lessard et al., 2018, 2019). Our findings suggest that accumulation of RPS14 in SDS fibroblasts is caused by a disruption in the function of the MAP7D1 protein that has a role in DNA damage repair (Dullovi et al., 2023); it is one of the causes of nucleolar stress in response to the p.R201W mutation and, thus, its inability to DNA repair. These findings also imply that the MAP7D1:p.R201W mutation contributes to SDS development by having a cumulative effect on cell cycle abnormalities, not only by disrupting the mitotic spindle assembly but also by triggering RPS14 accumulation. However, it is unknown which tumor suppressor proteins are activated by the MAP7D1:p.R201W mutation following accumulation of RPS14, which has never been associated with SDS, or which cell cycle-related molecular pathway underlying SDS it may trigger. Further investigations are, therefore, required.

In conclusion, we demonstrated that – like SBDS – MAP7D1 may have a dual role by regulating ribosomal proteins in addition to its microtubule stabilization function, and that the MAP7D1:p.R201W mutation disrupts interaction between microtubules and MAP7D1, affects microtubule stabilization and causes accumulation of the ribosomal protein RPS14, resulting in mitotic abnormalities in SDS cells. Given that mitotic aberrations are crucial in the development of pathogenic conditions, such as neutropenia, bone marrow failure and pancreatic insufficiency that are important for the etiology of SDS, we propose that the MAP7D1:p.R201W mutation contributes to SDS.

Overall, this study functionally identified a new *MAP7D1* gene mutation as probably being a causative gene for SDS. Approximately 10% of patients with clinically diagnosed SDS have no pathogenic variants identified in any known SDS-related genes. Although it is unclear why SDS occurs in these patients, it has been reported that pathogenic variants in other genes may also contribute to its development (Nelson and Myers, 2008). Thus, *MAP7D1* genetic testing in single-gene or multiple-panel screening for SDS diagnosis is critical for patients diagnosed with SDS with an unidentified genetic origin. However, the MAP7D1:p.R201W mutation has so far been limited to only one case of SDS. Genetic screening of *MAP7D1* and functional investigations of other possible pathogenic *MAP7D1* variants by using biological samples from other SDS patients are required to further validate the causality of *MAP7D1* for the development of SDS. Through further studies, more detailed investigation of mitotic abnormalities in the SDS pathogenesis, as well as identification of novel microtubule-associated gene mutations responsible for these abnormalities, would also contribute to the development of innovative treatment strategies for SDS. Currently, SDS is treated by using symptom-relieving therapies, such as blood transfusions, treatment with pancreatic enzyme supplements or granulocyte colony-stimulating factor (G-CSF) (Nelson and Myers, 2018; Han et al., 2023). However, there is presently no therapeutic intervention for the mitotic anomalies that result from microtubule instability, and which are considered to be one of the underlying causes of these clinical manifestations. At this point, microtubule-targeting agents (MTAs) may be novel treatment options for SDS. MTAs are chemical compounds that regulate microtubule dynamics by binding to tubulins, thereby stabilizing or destabilizing them (Steinmetz and Prota, 2018; Wordeman and Vicente, 2021), and have been used for a long time, to treat cancer and neurodegenerative diseases (Wordeman and Vicente, 2021). The utilization of MTAs to provide microtubule stability when treating SDS may also hold promise for the development of new therapeutic strategies. Further investigations of microtubule-associated processes and proteins – both of which have become significant contributors to the etiology of SDS – are essential for the advancement of alternative diagnostic and treatment strategies for SDS.

## MATERIALS AND METHODS

### Ethic approval and consent

Research protocols were approved by the Ethics Committees of Hacettepe University Non-Interventional Clinical Research and performed in compliance with standards set by the Declaration of Helsinki. All participants provided informed consent.

### Cell culture

Control and patient fibroblasts cultured from skin samples taken by needle biopsy from a healthy parent (father; +/− heterozygous) and the SDS patient (−/− homozygous), respectively, were kindly provided by Dr A. Çetinkaya (Department of Medical Genetics, Hacettepe University, Medical Faculty, Ankara, Turkey). Primary human fibroblasts, T98G glioblastoma cells (RRID:CVCL_0556) and HEK293T embryonic kidney cells (RRID:CVCL_0063) were cultured in Dulbecco's modified Eagle's medium (DMEM) (Gibco, Life Technologies, Carlsbad, CA, USA) supplemented with 10% fetal bovine serum (FBS) (Capricorn Scientific, Ebsdorfergrund, Germany) and 1% penicillin-streptomycin (Capricorn Scientific) at 37°C in 5% CO_2_ incubator. To maintain the culture, the cells were passaged with 2.5% trypsin (Gibco, Life Technologies) when they achieved a 70-80% confluency.

### Vector construction

The mutant MAP7D1 construct (MAP7D1_mut) was prepared from the primary fibroblast cDNA library of the SDS patient carrying the missense mutation c.601C>T. Briefly, full-length mutant MAP7D1 cDNA was amplified by PCR using Q5^®^ High-Fidelity 2× Master Mix (New England Biolabs, Ipswich, MA, USA) from the SDS fibroblast cDNA library of the patient. The sequences of the forward and reverse MAP7D1 primers used are 5′-ATAAAGCTTATGGAGAGCGGCCCGCGT-3′ and 5′-CGGAATTCAAGGACTTCTGTGACCTGCGGGGAC-3′, respectively. Then, the mutant MAP7D1 cDNA was cloned into the pcDNA3.1 (+)/myc-His A vector (Addgene, Watertown, MA, USA) into HindIII/EcoRI restriction sites. Wild-type MAP7D1 construct (MAP7D1_wt) was generated by site-directed mutagenesis of the MAP7D1_mut vector using the Q5^®^ Site-Directed Mutagenesis Kit (New England Biolabs) according to the manufacturer's instructions. The sequences of the forward and reverse site-direct primers used are 5′-GGAACGGCAGCGGCAGAAGCTCG-3′ and 5′-TCCAGGGCTGCACGGCGT-3′, respectively. The correct sequence of all constructs and the presence of the point mutation were verified by Sanger sequencing.

### Plasmid transfection and siRNA knockdown

For immunoblotting, T98G glioblastoma cells were plated at 4×10^5^ cells/well into six-well plates, while HEK293T embryonic kidney cells were plated at 7.5×10^5^ cells/dish on 60-mm cell culture dishes. Then, T98G cells and HEK293T cells were transfected for 24 h by using polyethylenimine (PEI) at ratios 1:4 and 1:3, respectively, with 4 µg of mock, MAP7D1_wt or MAP7D1_mut constructs. For pull-down analysis, HEK293T cells were plated at a density of 1.7×10^6^ cells/dish onto 100-mm cell culture dishes and transfected for 24 h with 10 µg mock construct or one of the MAP7D1 constructs (MAP7D1_wt or MAP7D1_mut) by using PEI at a 1:3 ratio. For immunocytochemistry analysis, T98G cells having large cytoplasm and allowing better visualization and MAP7D1 siRNA-treated HEK293T cells were plated at 1.5×10^5^ cells/coverslip or 1×10^5^ cells/coverslip on 0.01% poly-L-lysine (PLL)-coated coverslips in a 12-well plate. Then, T98G and HEK293T cells were transfected with 1.5 µg MAP7D1_wt or MAP7D1_mut construct using PEI in a 1:4 or 1:3 ratio for 24 or 48 h, respectively. For siRNA treatment, HEK293T cells having high transfection capacity and rapid growth rate (Tan et al., 2021) were plated at a density of 3.5×10^5^ cells/well on six-well plates. Cells were transfected for 96 h with 100 nM ON-TARGETplus Non-Targeting siRNA Pool (Cat#: D-001810-10-20) or 100 nM ON-TARGETplus Human MAP7D1 siRNA SMARTpool (Cat#: L-015538-01-0010) by using DharmaFECT2 transfection reagent (all reagents were from GE Healthcare Dharmacon, Lafayette, CO, USA).

### Cell synchronization

Cell synchronization was performed by using the double thymidine block method. Briefly, primary fibroblast cells and siRNA-treated and/or MAP7D1-construct-overexpressing HEK293T cells were plated on 0.01% PLL-coated coverslips into 12-well plates at a density of 1×10^5^ cells and 1.2×10^5^ cells, respectively. The next day, cells were treated with 2 mM thymidine (Calbiochem, San Diego, CA, USA) for 16 h in complete medium (DMEM, 10% FBS, and 1% penicillin-streptomycin). The resulting G1/S-enriched cells were washed twice with 1× phosphate-buffered saline (PBS) and released into the cell cycle in the complete medium for up to 8 h. Then, the cells were treated again with 2 mM thymidine for 16 h, followed by two washes with 1× PBS. Thereafter, complete medium was added again to the cells. For immunocytochemistry and proximity ligation assay cells were fixed every 30 min from 6 h until 9 h after thymidine release to arrest them at G2/M phase.

### Real-time PCR

Total RNA from primary fibroblast cells was extracted using the MN Nucleospin RNA kit (Macherey-Nagel, Düren, Germany). The extracted RNA was reverse transcribed by using the ProtoScript^®^ II First Strand cDNA Synthesis Kit (New England Biolabs) according to the manufacturer's instructions. Quantitative real-time PCR was performed with the LightCycler^®^ 480 Probes Master kit (Roche, Basel, Switzerland) by using a Roche LightCycler^®^ 480 Instrument II (Basel, Switzerland) according to the manufacturer's instructions. Probe and primers for *MAP7D1* used were the human MAP7D1 probe (UPL#65, Roche) and MAP7D1 primers (Forward: 5′-GGCTCCTCTGCATCACCTAC-3′ and Reverse: 5′-CCCAGGCCACTTCCATCTTT-3′). The human β-actin (ACTB) probe (Roche) was used as housekeeping control for relative expression analysis. Results were analyzed by using the ΔΔCT method (Livak and Schmittgen, 2001).

### Antibodies

Primary and secondary antibodies used for immunoblotting are as follows: MAP7D1 rabbit polyclonal antibody pAb (1:1000; Thermo Fisher Scientific, Cat#: PA5-44693, RRID:AB_2607602), Myc-tag mouse monoclonal antibody mAb (1:1000; Cell Signaling Technology, Cat#: 2276, RRID:AB_331783), Myc-tag rabbit mAb (1:1000; Cell Signaling Technology, Cat#: 2278, RRID:AB_490778), β-actin mouse mAb (1:1000; Cell Signaling Technology, Cat#: 3700, RRID:AB_2242334), β-actin rabbit mAb (1:1000; Cell Signaling Technology, Cat#: 8457, RRID:AB_10950489), β-tubulin rabbit mAb (1:1000; Cell Signaling Technology, Cat#: 2128, RRID:AB_823664), IRDye 800CW goat anti-mouse (1:15,000; LI-COR Biosciences, Cat#: 926-32280, RRID:AB_2814919), IRDye 800CW goat anti-rabbit (1:15,000; LI-COR Biosciences, Cat#: 925-32211, RRID:AB_2651127), IRDye 680RD goat anti-rabbit (1:15,000; LI-COR Biosciences, Cat#: 925-68071, RRID:AB_2721181). Primary antibodies, secondary antibodies, and dyes used for immunocytochemistry and proximity ligation assays are as follows: MAP7D1 rabbit pAb (1:100; Sigma-Aldrich, Cat#: HPA028075, RRID:AB_10603778), Myc-tag mouse mAb (1:250; Cell Signaling Technology, Cat#: 2276, RRID:AB_331783), Myc-tag rabbit mAb (1:200; Cell Signaling Technology, Cat#: 2278, RRID:AB_490778), γ-tubulin mouse mAb (1:150; Santa Cruz Biotechnology, Cat#: sc-51715, RRID:AB_630410), β-tubulin mouse mAb (1:100; Santa Cruz Biotechnology, Cat#: sc-58886, RRID:AB_793550), β-tubulin rabbit mAb (1:100; Cell Signaling Technology, Cat#: 2128, RRID:AB_823664), Tubulin rat mAb (1:1000; Abcam, Cat#: ab6160, RRID:AB_305328), RPS14 mouse mAb (1:200; Proteintech, Cat#: 67566-1-Ig, RRID:AB_2882780), Alexa Fluor-488 anti-Pericentrin antibody (1:250; Abcam, Cat#: ab270118), Alexa Fluor-488 conjugate goat anti-mouse IgG (1:250; Cell Signaling Technology, Cat#: 4408, RRID:AB_10694704), Alexa Fluor-488 conjugate goat anti-rabbit IgG (1:250; Cell Signaling Technology, Cat#: 4412, RRID:AB_1904025), Alexa Fluor-594 conjugate goat anti-mouse IgG (1:250; Cell Signaling Technology, Cat#: 8890, RRID:AB_2714182), Alexa Fluor-594 conjugate goat anti-rabbit IgG (1:250; Cell Signaling Technology, Cat#: 8889, RRID:AB_2716249), Alexa Fluor-546 conjugate goat anti-mouse IgG (1:250; Thermo Fisher Scientific, Cat#: A-11030, RRID:AB_2737024), Alexa Fluor-647 conjugate goat anti-rabbit IgG (1:250; Cell Signaling Technology, Cat#: 4414, RRID:AB_10693544), Alexa Fluor-647 conjugate goat anti-rat IgG (1:250; Cell Signaling Technology, Cat#: 4418, RRID:AB_1904017), DAPI (1:1000; Invitrogen, Cat#: D1306).

### Protein isolation and immunoblotting

For protein isolation, pellets obtained from primary fibroblast cells, T98G cells and HEK293T cells were dissolved in 1% NP-40 (Nonidet P-40) lysis buffer (150 mM NaCl, 1% NP-40, 50 mM pH8 Tris-Cl) and a protease inhibitor cocktail on ice and centrifuged at 13,000 ***g*** for 25 min at 4°C. The concentration of isolated proteins was determined with the Pierce™ BCA (bicinchoninic acid) Protein Assay Kit (Thermo Fisher Scientific, Waltham, MA, USA). For immunoblotting, the same amount of protein from each sample was separated by electrophoresis on a 10% SDS-polyacrylamide gel and transferred onto a nitrocellulose membrane (Santa Cruz Biotechnology, Dallas, TX, USA). The membrane was blocked with 5% non-fat dry milk in 1× TBS-T at room temperature for 1 h, first incubated at 4°C overnight with primary antibodies specific to the proteins of interest, followed by a second incubation with infrared fluorescent dye (IRDye) secondary antibodies at room temperature for 1 h. The membrane was washed three times with 1× TBS-T after each primary and secondary antibody incubation. Finally, the IRDye signal was detected with the Licor Odyssey CLx Near-Infrared Fluorescence Imaging System, and densitometric analyses were performed using Image Studio Lite (version 5.2, RRID:SCR_013715) and Adobe Photoshop CS5 (version 12.0, RRID:SCR_014199) software.

### Pull-down assay

Physical interaction between microtubules and MAP7D1, depending on the p.Arg201Trp mutation of the microtubule-binding domain of MAP7D1 was examined using the pull-down method with the MagneHis™ Protein Purification System Kit (Promega, Madison, WI, USA) according to manufacturer's instructions. Briefly, 600 µg lysate obtained from HEK293T cells overexpressing wild-type or mutant MAP7D1 were incubated with 35 µl MagneHis™ Ni particles and binding/wash buffer (100 mM HEPES, 10 mM imidazole, 500 mM NaCl) on a rotator at 4°C for 4 h. After incubation, tubes were placed on a magnetic stand to capture MagneHis™ Ni-Particles that were then washed three times with binding/wash buffer. Following, proteins bound to MagneHis™ Ni particles were eluted with 30 µl elution buffer (100 mM HEPES, 500 mM imidazole). Precipitated protein samples were separated using by western blotting, and 3% input samples were used as expression control.

### Immunocytochemistry

Primary fibroblast cells, T98G cells, and HEK293T cells were fixed with paraformaldehyde, mixed-aldehyde, or methanol. For paraformaldehyde fixation, cells were incubated with 4% paraformaldehyde at room temperature for 15 min and washed three times with 1× PBS. For mixed-aldehyde fixation, cells were incubated with fixation buffer [4% paraformaldehyde, 0.2% glutaraldehyde, 1× PHEM (PIPES, HEPES, EGTA, MgCl_2_) and 0.1% Triton X-100] at room temperature for 15 min. Then, the cells were washed twice with 1× PBS for 5 min and permeabilized with 0.25% Triton X-100 in 1× PBS at room temperature for 10 min. Following washing twice with 1× PBS, cells were treated with 1% sodium borohydride at room temperature for 15 min and washed again with 1× PBS, this step was repeated twice. For methanol fixation, cells were incubated in ice-cold methanol buffer (100% methanol and 0.1% Triton X-100) at −20°C for 5 min and washed three times with 1× PBS. After fixation, cells were blocked in blocking buffer [3% (w/v) bovine serum albumin and 0.1% Triton X-100 in 1× PBS] at room temperature for 1 h. Incubation with specific primary antibody was conducted at 4°C overnight. After primary antibody incubation, cells were washed three times with 1× PBS. Then, cells were incubated with Alexa Fluor-conjugated secondary antibodies and DAPI at room temperature for 1 h. Following the three washing steps with 1× PBS again, cells were mounted in equal volumes of ProLong™ Diamond Antifade Mounting Medium (Invitrogen, Carlsbad, CA, USA) to minimize the quenching of fluorescence signals during image capture. Cells were visualized using a TCS SP2 SE confocal microscope (Leica, Germany) with a 63× oil immersion objective or a Zeiss Axiovert A1 fluorescent microscope (Zeiss, Germany) with a 63× objective at single focal plane. Images were processed with Leica Application Suite X (LAS X) (version 1.4.5, RRID:SCR_013673), Zeiss ZEN (version 3.7, RRID:SCR_013672), and Adobe Photoshop CS5 (version 12.0, RRID:SCR_014199) software.

### Proximity ligation assay

Proximity ligation assay (PLA) was carried out to detect the interactions between γ-tubulin and wild-type or mutant MAP7D1. PLA was performed on fixed control and patient fibroblasts using the Duolink^®^ In Situ PLA^®^Probe Anti-Mouse MINUS kit (Sigma-Aldrich, St. Louis, MO, USA) as to the manufacturer's instructions. Briefly, fixed cells were blocked with Duolink blocking solution at 37°C for 1 h and incubated with appropriate primary antibodies diluted in Duolink antibody diluent buffer at 4°C overnight. After the washing step (two times with 1× wash buffer A), cells were incubated with plus and minus PLA probes at 37°C for 1 h. After two additional washes with 1× wash buffer A, cells were treated with ligation buffer at 37°C for 30 min and amplification buffer at 37°C for 100 min. Thereafter, cells were counterstained with DAPI and mounted on microscope slides in ProLong™ Diamond Antifade Mounting Medium (Invitrogen). Negative control incubations were used to confirm assay specificity and included omission of each primary antibody. Images were acquired using a TCS SP2 SE confocal microscope and LAS X (version 1.4.5, RRID:SCR_013673) software using a 63× oil objective in 3D mode at the *Z* position to cover the entire volume of centrosomes, generating 20 images per plane as raw data.

### Measurements

Fluorescent intensity for immunocytochemistry and PLA, the length between centrosomes, and the cross-sectional area of confocal images were measured using ImageJ (version 1.54, RRID:SCR_003070) software. To determine fluorescence intensity, the corrected total cell fluorescence (CTCF) was calculated using

CTCF=integrated fluorescence intensity−(area of selected cell×average background fluorescence). Background fluorescence was measured at five locations on the captured images and averaged. To measure length and cross-sectional areas, the known distance in the images, with the included scale bar, was set as the scale.

### Statistical analysis

Data were statistically analyzed using GraphPad Prism (version 8.0.1, RRID:SCR_002798) software. Statistical significance was determined using two-tailed Student's *t*-test (for two-group comparisons) or one-way and two-way ANOVA (for multiple-group comparisons) and is defined as follows: *P<0.05, **P<0.01, ***P<0.001, ****P<0.0001. n.s. (not significant), *P*>0.05. All data are presented as the mean±standard error of the mean (±s.e.m.).

## Supplementary Material

10.1242/dmm.052409_sup1 Supplementary information
