# Supplementary material for: A novel MAP7D1 mutation causes mitotic defects and RPS14 accumulation in Shwachman−Diamond syndrome patient cells
Source: Dis Model Mech. 2025 Aug 26;18(8):dmm052409. doi: 10.1242/dmm.052409 (PMC12421802; doi:10.1242/dmm.052409)
Supplement: Supplementary information [file dmm-18-052409-s1.pdf]

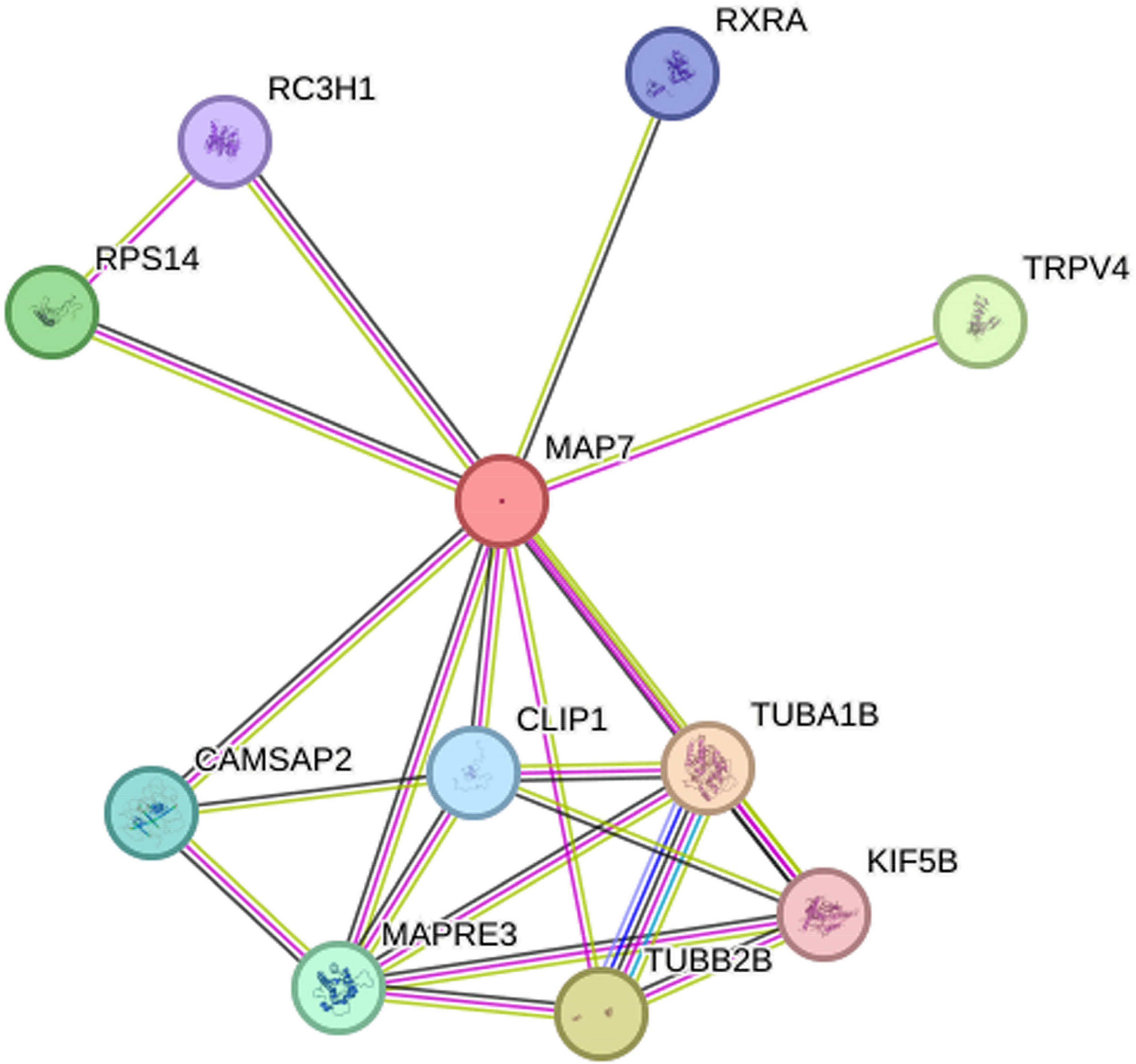

Fig. S1. Protein-protein interactions for MAP7 from STRING interaction database. Proteins are represented as nodes. Edges represent protein-protein associations. Cambridge blue: from curated databases, Violet: experimentally determined, Green: gene neighborhood, Red: gene fusions, Blue: gene co-occurrence, Reseda: text mining, Black: co-expression, Lila: protein homology.

Disease Models & Mechanisms • Supplementary information
